# *Notes from the Field*: E-cigarette, or Vaping, Product Use–Associated Lung Injury Cases During the COVID-19 Response — California, 2020

**DOI:** 10.15585/mmwr.mm6925a5

**Published:** 2020-06-26

**Authors:** Christina Armatas, Amy Heinzerling, Jason A. Wilken

**Affiliations:** ^1^Center for Healthy Communities, California Department of Public Health, Richmond, California; ^2^Epidemic Intelligence Service, CDC; ^3^Career Epidemiology Field Officer Program, CDC; ^4^U.S. Public Health Service, Rockville, Maryland.

In April 2020, during the early coronavirus disease 2019 (COVID-19) pandemic, eight patients hospitalized with e-cigarette, or vaping, product use–associated lung injury (EVALI) were reported to the California Department of Public Health (CDPH). Patients resided in five counties and were aged 14–50 years (median = 17 years); seven were aged <21 years. All hospitalizations occurred in April 2020, a median of 4 days (range = 4–13 days) after symptom onset. Four patients were admitted to an intensive care unit; two required mechanical ventilation. Nucleic acid testing for SARS-CoV-2, the virus that causes COVID-19, was performed on all patients at the time of hospitalization; all tests yielded negative results. Seven patients were tested two or more times, and lower respiratory tract specimens were tested from the intubated and mechanically ventilated patients. Patients met California and CDC EVALI case definitions, including negative respiratory pathogen testing and chest imaging findings consistent with EVALI (Box).* Health care providers first documented suspicion for EVALI in their notes on hospital days 1–8 (median = day 3), after testing for SARS-CoV-2 returned negative results. Six patients reported vaping tetrahydrocannabinol (THC)-containing products, one reported vaping only nicotine-containing products, and one did not specify products vaped. Seven patients had positive test results for THC on urine drug screen; one patient not tested by urine drug screen reported vaping THC. No epidemiologic links were identified among the patients. Two patients reported obtaining their vaping products from friends; six patients were not asked or did not disclose vaping product source. Recreational cannabis use is legal in California for adults aged ≥21 years. Products might have been acquired from informal or unlicensed sources by patients aged <21 years who reported THC product use.

BOX Provisional California Department of Public Health confirmed e-cigarette, or vaping, product use–associated lung injury (EVALI) case definition*

- Respiratory illness requiring hospitalization and
- Using an e-cigarette (vaping) or dabbing in the 90 days before symptom onset^†^ and
- Pulmonary infiltrate, such as opacities on plain film chest radiograph or ground-glass opacities on chest computed tomogram and
- Absence of respiratory infection on initial work-up: minimum criteria include the following negative tests: 1) SARS-CoV-2 nucleic acid test,^§^ and 2) respiratory viral polymerase chain reaction (PCR) panel, and 3) influenza PCR or rapid test, if local epidemiology supports testing, and 4) all other clinically indicated respiratory infectious disease testing (e.g., urine antigen for *Streptococcus pneumoniae* and *Legionella*, sputum culture if productive cough, bronchoalveolar lavage culture if done, blood culture, and human immunodeficiency virus–related opportunistic respiratory infections if appropriate) and
- No evidence in medical record of alternative plausible diagnoses (e.g., cardiac, rheumatologic, or neoplastic process).

* California has suspended use of the probable case definition, in which the patient has infection identified via culture or PCR but clinical team caring for patient believes this is not the sole cause of the underlying respiratory disease process; or patient has no evidence of pulmonary infection, but minimum criteria to rule out pulmonary infection not met (testing not performed).

^†^ Includes using an electronic device (e.g., electronic nicotine delivery system [ENDS], electronic cigarette, e-cigarette, vaporizer, vape(s), vape pen, dab pen, or other) or dabbing to inhale substances (e.g., nicotine, marijuana, tetrahydrocannabinol (THC), THC concentrates, cannabidiol, synthetic cannabinoids, flavorings, or other substances).

^§^ For critically ill patients requiring mechanical ventilation, a minimum of two negative SARS-CoV-2 tests are required, and at least one of the two specimens must be from a lower respiratory tract sample or bronchoalveolar lavage.

California identified 210 EVALI cases hospitalized during June 18, 2019–February 23, 2020,^†^ and 65 of 87 (75%) interviewed patients reported using THC vaping products obtained from informal sources (*1*). EVALI hospitalizations peaked nationwide in September 2019.^§^ Because of substantial declines in EVALI cases following their peak in September 2019, CDC discontinued the collection of EVALI case reports in February 2020. However, states could continue to collect data on EVALI cases. Because CDPH received reports of only four EVALI cases in February 2020, CDPH asked local jurisdictions to continue to report cases but discontinue active case interviews and follow-up at that time. The cases in April 2020 were the first reported to CDPH since February 2020 and the first since widespread transmission of SARS-CoV-2 was identified in California. It is unclear whether EVALI cases have continued to occur and were underreported or missed or whether these cases might represent the background incidence of EVALI as previously identified by CDC review of syndromic data (*2*). Because EVALI and COVID-19 signs and symptoms can be similar (e.g., cough, fever, and diarrhea),^¶^ (*3*) health care providers should maintain clinical suspicion for EVALI during the COVID-19 pandemic.

In May 2020, CDPH issued a health alert provisionally updating California’s EVALI case definition to require a negative SARS-CoV-2 nucleic acid test (Box) and suspending the probable case definition.** It is important that health care providers ask patients with symptoms consistent with EVALI, especially teenagers and young adults, about e-cigarette use, or vaping, during COVID-19 evaluations. CDPH urges everyone to refrain from using all e-cigarette, or vaping, products and recommends not using THC-containing products obtained from informal sources such as social contacts, online dealers, and unlicensed retailers.^††^

## Footnotes

*
https://www.cdc.gov/tobacco/basic_information/e-cigarettes/severe-lung-disease/health-departments/index.html .

†
https://www.cdph.ca.gov/Programs/CCDPHP/Pages/EVALI-Weekly-Public-Report.aspx .

§
https://www.cdc.gov/tobacco/basic_information/e-cigarettes/severe-lung-disease.html .

¶
https://www.cdc.gov/coronavirus/2019-ncov/symptoms-testing/symptoms.html .

** The CDC-Council of State and Territorial Epidemiologists case definition of EVALI has not been modified as of June 3 (https://www.cdc.gov/tobacco/basic_information/e-cigarettes/severe-lung-disease/health-departments/index.html#primary-case-def).

†† https://www.cdph.ca.gov/Programs/CCDPHP/Pages/Vaping-Health-Advisory.aspx.
